# Application of Geant4-DNA for simulating water radiolysis induced by Auger electron-emitting radionuclides

**DOI:** 10.1093/jrr/rrac105

**Published:** 2023-01-25

**Authors:** Daniel Adjei, Ngoc Duy Trinh, Mehran Mostafavi

**Affiliations:** Institut de Chimie Physique UMR8000, CNRS, Université Paris-Saclay, 91405, Orsay, France; ORANO, 125 Avenue de Paris, 92320 Châtillon, France; Institut de Chimie Physique UMR8000, CNRS, Université Paris-Saclay, 91405, Orsay, France

**Keywords:** radiolysis, Geant4 simulation, radiolytic yields, radionuclides

## Abstract

Auger-emitting radionuclides have potential application in targeted radiotherapy, particularly for metastatic cancers. This possibility, especially, is stemmed from their characteristic short-range (a few μm) in biological systems allowing localization of high dose within small tumours. To explore this potential application, a Geant4 Monte Carlo toolkit has been employed to simulate the energy deposition of different radionuclides in a water model. The Geant4 Monte Carlo toolkit has model packages to simulate the interaction of radiation with matter and with diverse applications such as studies in science and medicine. In this study, the Geant4-DNA package was used to simulate the radiolytic yields induced by some Auger electron-emitting (AE) radionuclides including; I-131, I-125 and Pd-103, In-111, Ru-97 and Rh-103 m in water model. The results showed that the transient yield of the radiolytic species is characterized by the kinetic energies of the emitted electrons. It was observed that almost all the radionuclides, except I-131, deposited more energy in their proximity thereby inducing a high density of spurs to interact in a short time. It is, therefore, important to consider the kinetic energies of the emitted particles in choosing a radionuclide for specified targeted radiotherapy. This means that apart from their toxicity, compatibility with chelator and carrier molecules, and method of production, we can predict radionuclides such as In-111, Ru-97, Pb-103 m and I-125 could be relevant for targeted radiotherapy for the treatment of metastasis lesions, or tiny tumours at the cellular level, and tumours after surgical resection.

## INTRODUCTION

Treatment of solid cancers over several decades has been managed by the combination of surgery, chemotherapy and radiotherapy. Radiotherapy may be the main treatment or may be used to assist other treatment techniques. External beam radiotherapy techniques such as conformal radiotherapy, pencil beam radiotherapy, etc. have been effectively employed for the treatment of solid cancers. However, external beam radiotherapy has not been used effectively for the treatment of metastasis lesions or tiny tumours at the cellular level [1]. This is because it is practically difficult, if not impossible, to focus an external radiation beam onto such tiny tumours and to spare at the same time the nearby healthy tissues, especially with the most popular electron and photon external radiotherapy. Proton and hadron therapy with a ^12^C beam should help to focus better on the tumour thanks to the Bragg peak effect. However, due to the high cost and the complexity and rarity of proton and hadron therapy accelerators, the number of patients treated with these techniques is limited (~4000 patients annually in Europe [2]). Furthermore, focusing on tiny tumours is still very complex for proton and hadron therapy. As such selective techniques such as radionuclide targeted therapy are more desirable. Based on their promising application for targeted radiotherapy, some carrier molecules have been developed to incorporate them for treatments such as malignant, lymph, etc. The main aim is to deliver a high radiation dose to a specific site of tumour or cells to spare healthy tissues [3–6]. To achieve this specificity, the incorporated radionuclides should emit particles that have a short range in the order of the size of the cell or the tumour size of interest [7]. However, different radionuclides emit different types of radiation with different energies. Therefore, in choosing a radionuclide for targeted radiotherapy, the emitted particles need to be considered in addition to their toxicity, compatibility with a chelator and cell targeting, as well as the mode of production (extraction from mineral/nuclear waste, production by irradiation in nuclear reactor/accelerator).

Radionuclides that emit particles such as Auger electrons (AEs) [8–9] by electron capture are suitable for personalized targeted radionuclide therapy [10–11]. These ionizing radiations have the potential to cause damage to biological systems through ionization or the breaking of important bonds. This may be through a mechanism called direct or indirect effects. The latter is, particularly, common with particles of high linear energy transfer (LET) and can potentially cause direct damage to cellular components [12]. The contribution of direct ionization is believed to be much higher for high LET particles as compared to low LET particles used in external radiotherapies such as X-rays and high-energy electrons. High LET particles such as AEs typically have a shorter range, 1 nm to 30 μm in water, depending on their kinetic energies. Due to the short range of these particles, AEs deposit much energy around the decaying site and thereby inducing detrimental effects such as DNA single- and double-strand breaks [13].

For the indirect effect, emitted decay particles may also interact with cellular medium, especially water, leading to discrete deposition of energy. This interaction may lead to excited water (H_2_O^*^) or ionized water(H_2_O^•+^). The ejected electron (noted as secondary electron) from the ionized water may have enough kinetic energy to cause further ionizations until it reaches thermalization at thermic energy of ~25 meV. The excited or ionized water molecule also undergoes a dissociative or proton transfer processes within a few tens of femtoseconds initiating a variety of reactive intermediates that react and disappear at different rates. The products of these processes such as ^•^OH, H^•^, H^+^ and e^−^_aq_ are considered the first species formed during water radiolysis. They may also undergo further reactions with the neighbouring network of water molecules, playing the roles of reactant and solvent by dipolar orientation in solvation processes to form highly oxidative species such as H_2_O_2_. Most of these radical species are detrimental to DNA by breaking bonds and rendering it damaged in a mechanism known as an indirect effect.

To estimate the relative contribution of indirect and direct damage to DNA due to ionizing radiation, several cellular and animal models have been employed [14–16]. However, with radionuclides, it is practically difficult to test on cellular and animal models due to their complex toxicity. Simulation tools could, therefore, be used to complement such studies. With these tools, it is possible to describe the basic interaction of radiation with matter and the principles of subsequent chemical reactions which may lead to biological damage [17].

Therefore, numerous Monte Carlo track structure codes such as Geant4-DNA [13, 18–22] of Geant4 Monte Carlo toolkit [23–24] ; PARTRAC [25] ; KURBUC [26] and many others have been modelled to simulate physical interactions and water radiolysis products [27]. Track structure codes ensure the accuracy of their length scales of interactions which have a biological interest. To be able to predict the biological consequences of radiation, it is important to simulate secondary electrons down to the excitation (or ionization) threshold of the medium which is around 10 eV for liquid water. Thus, track codes provide a detailed treatment of cell interactions using single-scattering models and they offer an appropriate resolution of small biological targets. One such Monte Carlo track code with the capacity to simulate the physical, physicochemical and chemical stages and with a distinct advantage of being open source making it accessible by many users and developers is the Geant4-DNA Monte Carlo package. This Geant4-DNA package which is incorporated in the Geant4 Monte Carlo toolkit makes use of a track structure to describe the step-by-step physical electromagnetic interactions of particles and some ions with liquid water. The details of the Geant4-DNA model are described in Incerti *et al.,* Incerti, Kyriakou *et al.* and André *et al.* [28–30]. Here, we simply highlight a few of the tools in this package which is crucial to this study.

Geant4-DNA can operate at low energy (~10 eV) interaction that is governed by significant theoretical and experimental studies. Since the release of the Geant4-DNA, it has been much improved and adopted as an investigational tool for radiolysis and radiobiology studies [20, 28, 31–32]. This code is benchmarked to other track structure Monte Carlo codes and, where available, against reference experimental data. The Geant4-DNA physics models and radiolysis modelling functionalities have already been described in detail in the literature [33]. Some works have been done to evaluate the yields of typical radiolysis products (such as e_aq_^−^, ^•^OH and H_2_O_2_) under irradiation using the Geant4-DNA [34–35] . In addition, advanced work has been done to improve the Geant4-DNA package for simulating damage by radiation-induced in some specific biological targets [36–38].

In this work, the Geant4 Monte Carlo toolkit is used to simulate the radiolytic effect of Auger-emitting radionuclides. It is also used to simulate the energy deposition pattern of the full decay of selected radionuclide. This preliminary step will elucidate the choice of radionuclide for application in targeted radiotherapy. Further work is underway utilizing Geant4-DNA to simulate DNA damage by radionuclides.

## MATERIALS AND METHODS

A 20-core Intel Xeon computer of 3.7 GHz CPU (120 GB DDR4 RAM, 3.2 GHz) with Ubuntu 22.04 LTS version operating system was used to perform these simulations. The Geant4 Monte Carlo toolkit (version 10.06.p02) was employed to simulate the decay of radionuclides to elucidate their pattern of energy deposition while the Geant4-DNA package was used to simulate radiolytic species induced as a result of the decay of these radionuclides. The NIST ‘G4_WATER’ and ‘G4_AIR’ materials were used to define liquid water of density 1 g/cm^3^ and air, respectively. In this study, six radionuclides of potential interest for targeted radiotherapy were used, including I-125, I-131, Pd-103, In-111, Ru-97 and Rh-103 m, and were selected as radiation sources.

### Simulation of energy spectrum by Auger-emitting radionuclides

The spectra emission of each radionuclide was computed for electron/positron and photons. Here, a simple homogeneous air medium was constructed and the Geant4 G4radioactiveDecay, G4Transportation processes and relevant particles were defined in a physics class and a full radioactive decay chain was activated to track the decay of all daughter radionuclides and particles. At each decay, the energy spectrum was computed.

### Simulation setup for energy deposition by Auger-emitting radionuclides

The radioactive decay and the subsequent energy deposition in water were simulated by the Geant4 Monte Carlo toolkit. This simulation was performed about the Geant4 extended examples of ‘TestEm7’ and ‘rdecay01’. This is because the Geant4 offers exemplary applications which are available in open access to serve as references for users. Here, a cube of water of geometry 1 mm^3^ was constructed in the Geant4 detector construction class, and a set of 10^6^ radionuclides was launched on the side of the box. The energy deposited along the trajectory of the incident particle was computed per micrometre depth in water. The radionuclide of interest was defined by specifying the atomic number, mass number and excitation energy in the Geant4 G4ParticleGun class. To process the decay of the radionuclides and their subsequent emissions, the Geant4 default radioactive decay constructor was added to the physics class. This decay model handles alpha, beta, positron, anti-neutrino, de-excitation gamma rays, etc. as well as follows all the descendants of the decay chain. Geant4 default radioactive decay constructor employs the branching ratio and the decay scheme data of the well-known Evaluated Nuclear Structure Data File (ENSDF). The use of such evaluated data is important for the validation of the physics results of the experiments. The atomic relaxation model was invoked in the physics class to account for Auger emission. In addition, the electromagnetic physics constructor known as ‘G4EmStandardPhysics_Option3’ was implemented to simulate the emitted particles through the volume of liquid water. This constructs all electromagnetic processes as well as the corresponding particles and is recommended for medical and space science applications. By tracking each radiation emitted, the energy deposited along their trajectories was computed.

Under similar simulation conditions, another geometry consisting of a cube of water 30 cm × 30 cm × 20 cm with a sphere of water placed in the centre of it was constructed. Supplementary Figure S1 shows the geometry used to simulate the calculation of the energy deposition by radionuclides. The inner-sphere volume is considered the target volume while the outer volume represents an untargeted volume. The radius of the target volume was varied for each simulation condition to compute how much dose is deposited in the targeted volume in comparison with the untargeted volume. A set of radionuclides is placed in the centre coordinates of this sphere, and from simulation, the dose (Gy) delivered by a radionuclide in the sphere of a target volume is calculated as $D=E/m,$ where $D$ is the dose in gray (Gy), $E$ is the energy (J) deposited by a radionuclide in a sample of mass in Kg ($m$) calculated based on the radius of sphere and density of water.

### Simulation of radiolytic yields induced by Auger-emitting radionuclides

In this section, the Monte Carlo simulation of the radiolytic yields induced by radionuclides of interest has been carried out by employing the Geant4-DNA extension of the Geant4 toolkit. The radiolytic yields were recorded for the different radionuclides of interest when placed in liquid water of geometric size of 100 μm × 100 μm × 100 μm. The Geant4-DNA package comes with examples that demonstrate its functionality for the simulation of physical, physicochemical and chemical stages of water radiolysis. It has a unique capability of linking the pre-chemical stage of the water radiolysis with the physical and chemical stages. Here we employed the Geant4 decay constructor to process the decay of the different radionuclides. The ‘G4EmDNAPhysics_option2’ constructor which is able to simulate step-by-step the physical interactions of electrons down to very low energies (~10 eV) in liquid water was employed. The ‘G4EmDNAChemistry_option3’ which is also very accurate for the simulation of the physicochemical and chemical stages of an irradiated water medium up to one microsecond after irradiation was also used [22].

With these constructors employed in our simulation code it was possible to simulate the initial radiolytic yields called the G-values, which are the number of molecular species normalized per 100 eV of deposited energy. The simulation employs all the possible reactions and products of water radiolysis as described in the literature [28]. Thus, after energy deposition, the ionization and excitation events are assumed to lead to the formation of the species of H_2_O^+^, H_2_O^*^ and subexcitation electron(e_sub_), which later dissociates into H_aq_^+^, and •OH as well as the subexcitation electron [39–41].

## RESULTS AND DISCUSSION

### The energy deposition trajectory in water by the decay of radionuclides

**Fig. 1:**
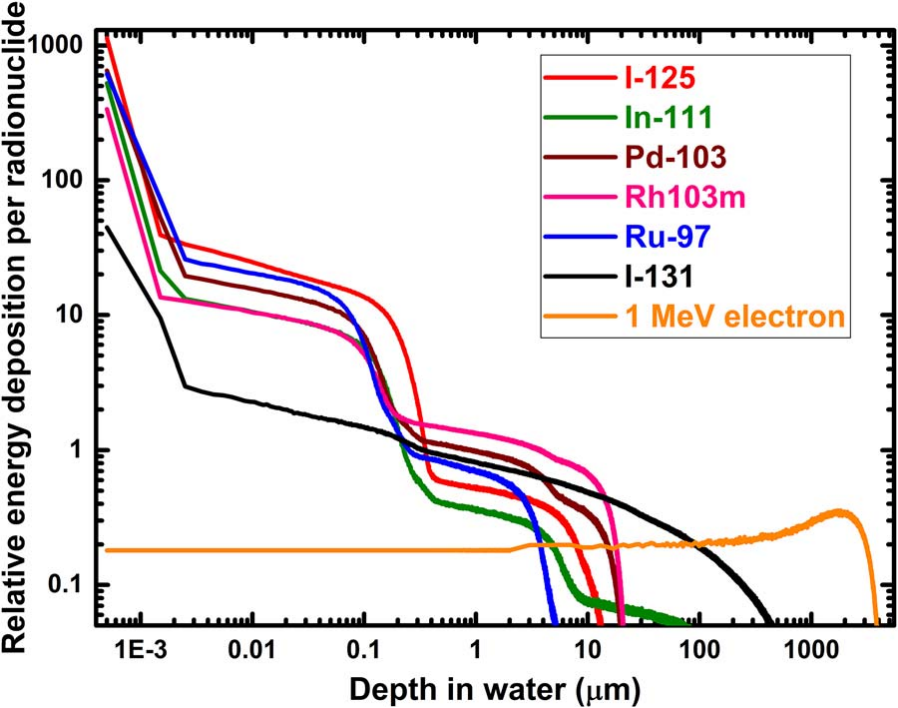
Simulation of energy deposition by Auger-emitting radionuclides and 1 MeV electron along the trajectory of the incident particle in water.

**Fig. 2:**
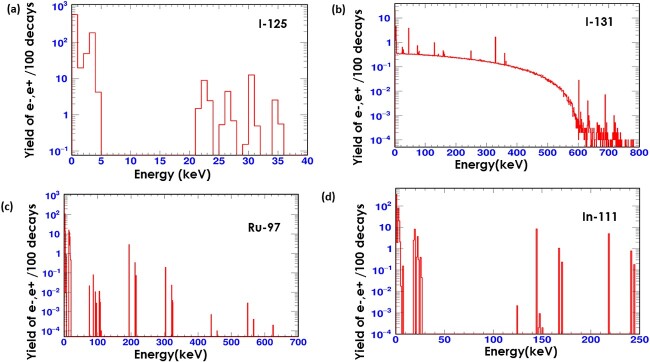
Geant4 simulation of the yield of e−/e + per every 100 radionuclide decays of (a) I-125, (a) I-131, (c) Ru-97, (d) In-111.

**Fig. 3:**
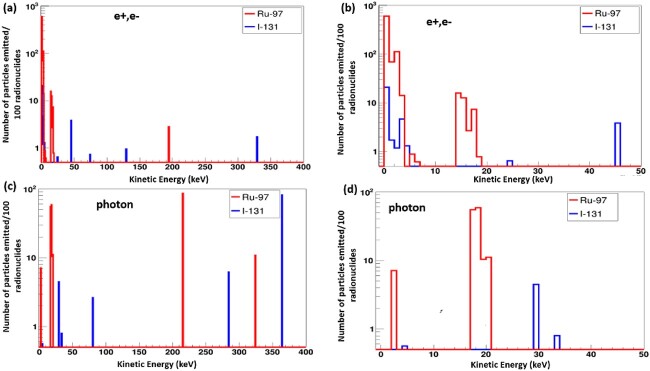
Geant4 simulation of the yield of e−/e + and of photons per every 100 radionuclide decays of I-131 vs Ru-97. (a) is the expanded yield of the electron spectrum up to 400 keV and (b) is the electron spectrum up to 50 keV of I-131 vs Ru-97. (c) and (d) are the yields of photon spectra up to 400 keV and up to 50 keV, respectively of I-131 vs Ru-97.

One key consideration for the selection of radionuclide for targeted radiotherapy is the range of the emitted particles. The simulation result of the depth of energy deposition in water by Ru-97, Pd-103, Rh-103 m, In-111, I-125 and I-131 is reported in Fig. 1. The results show that the trajectory of the energy deposition of each radionuclide is different for each radionuclide mainly due to the kinetic energies of the emitted radiation. An example of the simulation emission spectra of some of the selected radionuclides is presented in Fig. 2. Thus, the depth to which each radionuclide will deposit its energy is dependent on the emission spectrum of that radionuclide. Low energy emitting radionuclides deposit a high dose in the vicinity of the radionuclide. These low-energy electrons are mainly from Auger emissions. The low-energy AEs have a very short range of energy deposition, and so deposit much more of their energy at the site of the decay. The dominance of the AEs has significant implications for the distribution of energy that is deposited in the vicinity of the radionuclide. This is evident from the energy spectrum of each radionuclide. It is evident that, for example, the lower energy AEs emitted by Ru-97 compared with the higher energy beta emission from I-131 will induce much energy deposition in a very small water medium than for I-131. Figure 3 shows a comparison of the simulated emission spectra of I-131 and Ru-97. I -131 has a broader spectrum of electrons and photons of higher kinetic energies and deposits energy as far as 1 mm depth in water, larger than a typical human cell. However, radionuclides such as I-125, Ru-97, Pd-103, Rh-103 m and In-111 show a range of energy deposition in water within a few hundreds of micrometres. Thus, these Auger isotopes located inside the cell or nucleus will deposit much of their energy in it with minimal energy deposition in the nearby cells or tissues. The different range exhibited by each radionuclide is vital in choosing a radionuclide for a particular size of tumour or cell in targeted radionuclide therapy.

### Energy deposition per unit sphere of water

**Fig. 4:**
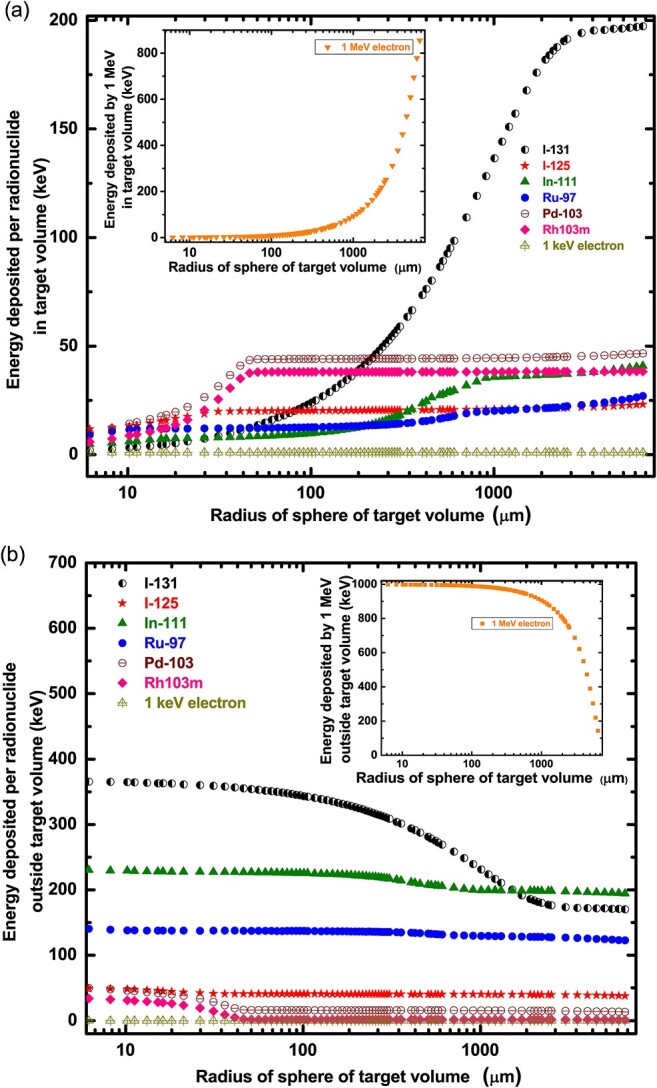
Simulation of (a) energy deposition per radionuclide in an area of a spherical unit radius target volume (liquid water). The insert is the energy deposition by 1 MeV electron for the same geometry as was selected for the radionuclides for comparison. (b) The deposited energy in the outside remaining volume for a varying spherical radius. The insert is the energy deposition outside the target volume by 1 MeV electron for the same geometry as was selected for the radionuclides for comparison.

In the same way, the energy deposition in the water model is estimated based on varying the radius of the sphere of water (Fig. 4). In Fig. 4a, the highly energetic electrons and photons emitted by I-131 deposit energy in large spheres around 1 mm. Thus, beyond a sphere of 1 mm radii, I-131 still deposits some energy as shown in Fig. 4b. The remaining radionuclides deposit almost their full energy within a few tens of a micrometre inside a target volume. Radionuclides such as I-125, Pd-103 and Rh-103 m, deposit most of their energy in small volumes (< 100 μm) of targets with minimal dose to nearby volumes. Radionuclides such as Ru-97 though leave some energy deposition in a long range but are not as significant as shown by I-131. The energy deposition in a short range by each radionuclide could be considered in selecting radionuclide irradiation of targeted cells or small tumours. Localizing Auger emitting radionuclides inside a typical human cell of about 10 μm in diameter, or for smaller tumour cells could be suitable for radiotherapy while limiting damage to nearby healthy cells or tissues. This property of Auger emitting radionuclides makes them attractive for targeted radiotherapy such as metastatic tumours. Radionuclides that emit AEs also release photons. These photons penetrate deeper into cells and tissues. Due to this diverse radiations and energy deposition distance, the energy deposited in the remaining volume, as shown in Fig. 4b, should be taken into consideration when deciding on the choice of radionuclide for targeted radiotherapy.

The dose deposited by the decay of the different radionuclides inside a target volume is presented in Table 1. For a smaller volume such as 10 μm, Pd-103 would deposit a high dose of ~543 μGy just a little above I-125. However, Pd-103 emits electrons of relatively higher kinetic energies which would cause dose deposition also in untargeted volumes. All other radionuclides deposit relatively a minimum amount of energy within a 10 μm radius of a target volume. For larger volumes, I-131 can deliver its maximum dose in it.

**Table 1:** Dose deposition in target volume of different radii

Dose (μGy/decay)						
Radius of sphere	10 μm	20 μm	50 μm	100 μm	1000 μm	6500 μm
I-131	122.00	29.15	4.16	0.92	1.22E-4	4.44E-7
I-125	503.00	83.80	6.16	0.78	8.02E-4	3.23E-6
In-111	232.00	36.50	2.59	0.78	1.37E-3	5.69E-6
Ru-97	443.00	57.40	3.75	0.48	7.71E-4	3.77E-6
Pd-103	543.00	105.32	13.50	1.69	1.70E-3	6.50E-6
Rh-103 m	336.00	76.90	11.60	1.46	1.46E-3	5.34E-6
1 MeV electron	35.3	9.1	1.41	0.35	3.58E-3	1.19E-4

### Radiolytic yields by Auger-emitting radionuclides

**Fig. 5:**
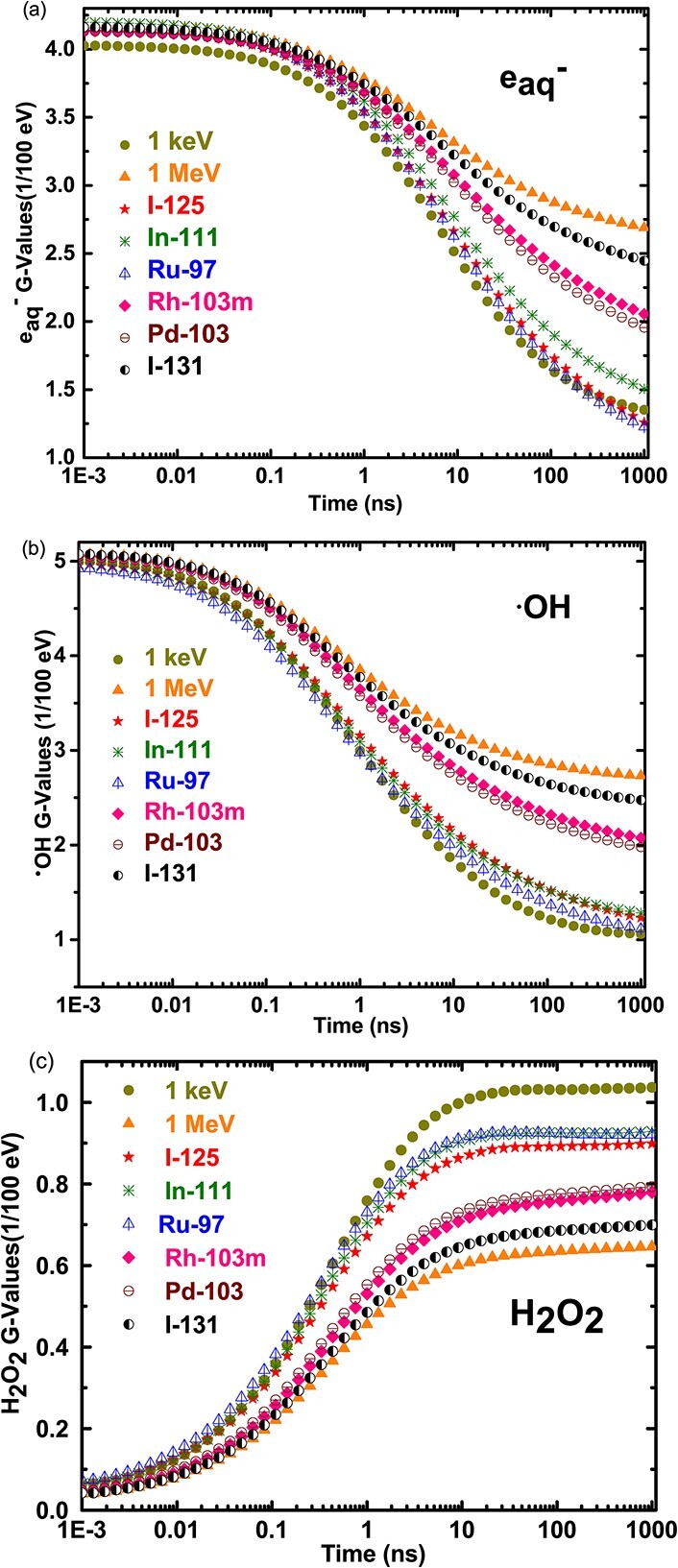
Time-dependent G-values of (a) e_aq_^−^, (b) ^•^OH and(c) H_2_O_2_ as a result of the interaction of the decay products of various radionuclides, 1 keV- and 1 MeV-(selected as low LET radiation) electron with liquid water.

The time-dependent yields of several species induced in water due to the decay of Auger emitting radionuclides are simulated with the Geant4-DNA package. However, we report here only a few of these species of biological interest. Figure 5 shows the time-dependent yields of species such as e_aq_^−^, H_2_O_2_ and ^•^OH induced by the decay of the different radionuclides under consideration.

The result shows that the initial G-values of all the radionuclides (I-125, I-131, In-111, Pd-103, Rh-103 m and Ru-97) for hydrated electron and the hydroxyl radical at 1 ps are around 4.2 × 10^−7^ mol^-J^ and 5.1 × 10^−7^ mol^-J^, respectively which are very close to that found experimentally for low energy electron beam of a few MeV [36, 42–44]. These values decrease until 100 ps, and then a distinct difference shows up for the different radionuclides at later time. It becomes more significant after 1 ns where it is observed that the G-values of these primary species were the highest for I-131; followed by Rh-103 m and then Pd-103, In-111; followed by I-125 and Ru-97. This variation is inferred from the different emission spectra of the different radionuclides.

It is shown that the decay of radionuclides induces the emission of particles and photons of energies ranging from low to high. This mix of electrons/photons of different energies results in different LETs. It can be deduced that the varying G-values at a later time are associated with the effect of a mix of LET induced by a radionuclide. For example, I-131 which emits high energetic electrons exhibit a high G-Value of the solvated electron at a longer time as compared to I-125 whose decay induces a smaller G-value of the solvated electron. This was tested by comparing the time-dependent G-values of electrons of specific energies such as 1 keV and 1 MeV as shown in Fig. 5.

The first species formed such as e_aq_^−^ and ^•^OH during water radiolysis starts with diffusion and reaction with the neighbouring network of water molecules. In highly concentrated radicals, inter-radical recombination occurs thereby changing the G-values of species present at this time. Here, the large spectra emission of a radionuclide induces different spatial energy deposition and therefore changes the inter-radical recombination and affects the diffusion of radicals with time. Therefore, the variation of the G-values at later time can be attributed to the influence of the energy deposition pattern which is reflected by the LET of the emissions from the decay of a radionuclide. High energetic radiation causes spatially distributed radiation spurs and that decreases the probability of radical-radical reactions. In contrast with low energetic radiation, the radiation spurs are dense causing faster decay of radicals due to the high probability of radical-radical reactions. Thus, radionuclides that emit mainly low energetic electrons induce high dense radical species to induce radical-radical interactions or radical-molecule reactions which has biological consequences. However, for highly energetic electron and photon emitters, the G-values decline slowly due to their greater spatial dose distribution. Thus, the more densely the radical species, the more probable it may interact with biological systems to induce damage or form other products that can be more damaging.

Hydrogen peroxide is known to transmit cellular signals that can cause oxidative folding of exported proteins, and in excess, be damaging to cells and tissues. In this study, we present the G-values of hydrogen peroxide which is shown to increase with time for the different radionuclides and get to a plateau at a late time. This is formed as a result of the combination of OH radicals. For this reason, a solution with a high density of ^•^OH produced is favoured by a high rate of formation of hydrogen peroxide. It is well-known that for high-LET such as AEs, molecular species (such as H_2_ and H_2_O_2_) are favoured by an intensified recombination of radical species (H^•^, ^•^OH and e_aq_^−^)^45^. By this mechanism, high-LET radiation inducing a high dose at the site of the decay leads to the subsequent production of a high density of radicals [45–47]. Therefore, it can be inferred that the high G-value (H_2_O_2_) of ^111^In compared with that of 1 MeV electron is due to low-energy AEs emitted by ^111^In. An Auger emitting radionuclide which has the potential to induce more hydrogen peroxides is also attractive for targets such as DNA and the cell membrane.

## CONCLUSIONS

The Geant4 Monte Carlo toolkit which offers the possibility to simulate the passage of particles through matter; applied in various fields of science including nuclear physics, medical physics and many more, is used to simulate the energy deposition in water by some Auger emitting radionuclides and the subsequent radiolytic yields. It is observed that radionuclides that emit cascades of low-energy AEs deposit much of their energy in a few nanometers to a micrometre range at the proximity of the decay site within the range of typical cell size. They are, therefore, suitable for targeted radiotherapy. For example, I-125 which emits electrons of kinetic energy in the range of a few eV to 35 keV could penetrate up to ~10 μm depth in water. Radionuclides such as Pd-103 (emitting electrons/positrons of kinetic energies of 7.266 eV - 271.7 keV) and Ru-97 (emitting electrons/positrons of kinetic energies of 0.001561 meV - 733.3 keV), can also deposit much of their energy nearby (20 μm) from their low-energy electrons although they produce some electrons of higher kinetic energies that can deposit their energy at deeper depth in water. Among them, I-131 was found to deposit much of its energy in larger volume targets. Indeed, when decaying in proximity to a biological molecule like DNA, the AEs emitted by I-125 would deposit much more dose in it than will be deposited by the longer-range particles emitted by I-131.

Radiolytic yields provide fundamental information on the species induced due to the presence of a particle in water model. The simulation of the yields induced by radionuclides in water provides important information on the selection of radiotherapy for targeted radiotherapy by considering also other factors such as toxicity, half-life, etc. In this work, we report also for the first time the average G-values that can be expected from the decay of some radionuclides. It is evident from the reported results that the time-dependent radiolytic yields at a later time depend on the type of radionuclide which is related by their emission spectra. In biological systems, there are two major reactive oxygen species, superoxide radical and hydroxyl radical. In the simulation, we show that low-energy electron-emitting radionuclides such as I-125, Ru-97 and In-111 exhibit fast decreasing G-values of hydroxyl radical. The degradation of the hydroxyl radical is a sign of oxidation that is predominantly of biological consequences such as oxidative stress or DNA damage. With highly dense ‘spurs’ of hydroxyl radicals induced by I-125, Ru-97 and In-111 leading to an increasing G-values of H_2_O_2_ is a sign of toxicity that these radicals can induce in biological systems. In general, we conclude that low-energy Auger-emitting radionuclides induce radiolytic processes which may cause detrimental biological consequences. These short-range emitted particles also play important role in targeting biological systems at the micrometre scale. Given these, this work provides fundamental information that is of relevance in choosing radionuclides for targeted radiotherapy, where selectivity is a more promising treatment modality. It is also important to emphasise that the Geant4-DNA Monte Carlo toolkit is a powerful tool to simulate not only the physical, physico-chemical and chemical stages of water radiolysis but also be combined with geometric models of biological targets, such as DNA, to assess direct and indirect early DNA damage. Therefore, the perspective of this work is to extend to the simulation of the indirect and direct DNA damage yields due to the presence of the radionuclides.

## DATA AVAILABILITY

We confirm that all data related to this article is available for accessibility whenever required.

## CONFLICT OF INTEREST

The authors declare they have no conflicts of interest.

## FUNDING

This study received a financial support from ORANO in the framework of R&D collaboration contract ‘Étude théorique de dommages induits à l’AND par des radionucléides émetteurs des électrons Auger par Geant IV’ between Orano and ICP Orsay.

## Supplementary Material

Appendix_rrac105
