# Supplementary material for: Application of Geant4-DNA for simulating water radiolysis induced by Auger electron-emitting radionuclides
Source: J Radiat Res. 2023 Jan 25;64(2):369–78. doi: 10.1093/jrr/rrac105 (PMC10036101; doi:10.1093/jrr/rrac105)
Supplement: Appendix_rrac105 [file appendix_rrac105.docx]

Appendix


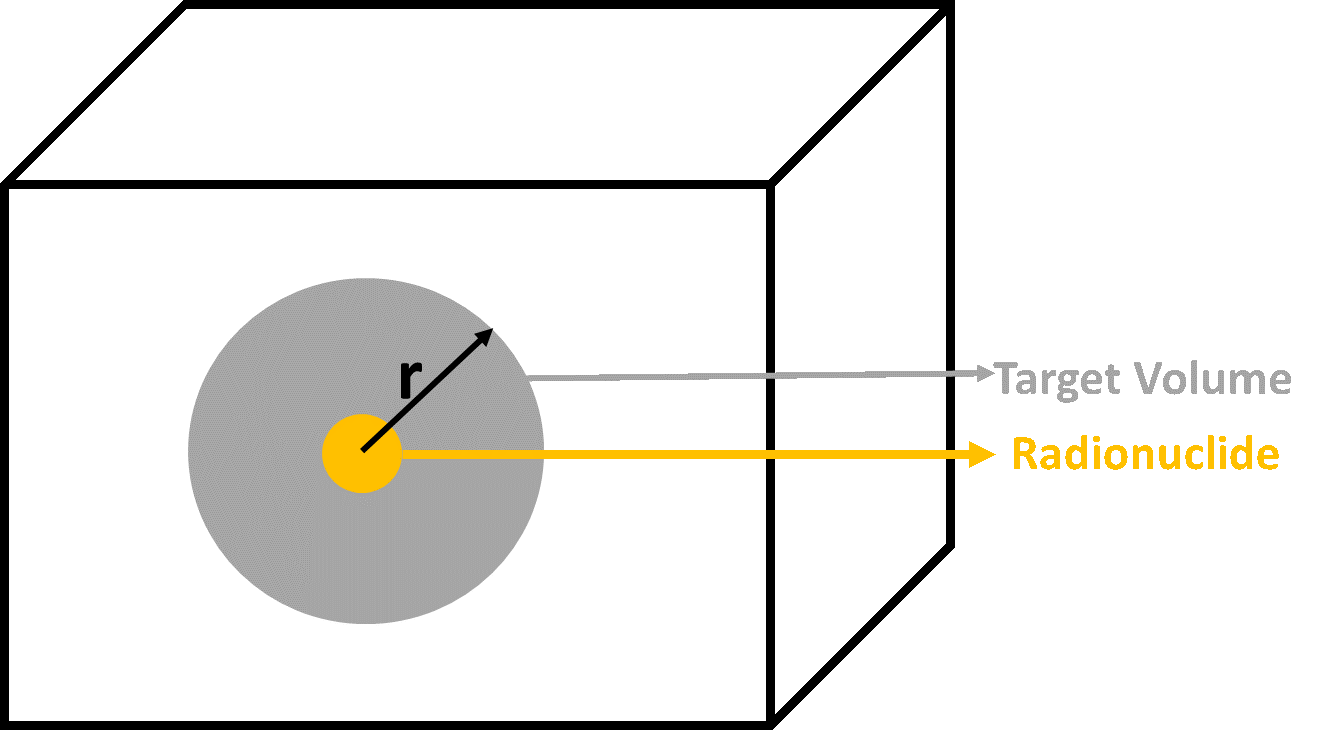


Figure SI: Geometrical setup for the simulation of energy deposition in a unit area of sphere of radius (r) in a water model. The 30×30×20 cm3 is considered as the full biological system.
